# The use of a non-biological, bridging, antiprotrusio cage in complex revision hip arthroplasty and periacetabular reconstructive oncologic surgery. Is still today a valid option?: A mid/long-term survival and complications’ analysis

**DOI:** 10.1007/s00402-021-03929-6

**Published:** 2021-05-24

**Authors:** Matteo Innocenti, Francesco Muratori, Giacomo Mazzei, Davide Guido, Filippo Frenos, Ersilia Lucenteforte, Rodolfo Capanna, Domenico Andrea Campanacci

**Affiliations:** 1 Department of Orthopaedic Oncology and Reconstructive Surgery, Azienda Ospedaliero-Universitaria Careggi, University of Florence, Largo Palagi 1, 50139 Florence, Italy; 2 Unit of Medical Statistics, Department of Clinical and Experimental Medicine, University of Pisa, Pisa, Italy; 3 Department of Orthopaedic and Trauma Surgery, Azienda Ospedaliera Universitaria Pisana, Pisa, Italy

**Keywords:** Antiprotrusio cages, Revision total hip arthoplasty, Periacetabular bone losses, Pelvic discontinuity, Primary pelvic bone tumors, Periacetabular bone metastases

## Abstract

**Introduction:**

Burch–Schneider-like antiprotrusio cages (B-SlAC) still remain helpful implants to bridge severe periacetabular bone losses. The purpose of this study was to evaluate outcomes and estimate both cages’ failures and complication risks in a series of B-SlAC implanted in revision of failed total hip arthroplasties (THA) or after resection of periacetabular primary or secondary bone malignancies. Risk factors enhancing the chance of dislocations and infections were checked.

**Materials and methods:**

We evaluated 73 patients who received a B-SlAC from January 2008 to January 2018. Group A, 40 oncological cases (22 primary tumors; 18 metastases); Group B, 33 failed THAs. We compared both Kaplan–Meier estimates of risk of failure and complication with the cumulative incidence function, taking account the competing risk of death. Cox proportional hazards model was utilized to identify possible predictors of instability and infection. Harris hip score HHS was used to record clinical outcomes.

**Results:**

Medium follow-up was 80 months (24–137). Average final HHS was 61 (28–92), with no differences within the two groups (*p* > 0.05). The probabilities of failure and complications were 57% and 26%, respectively, lower in the oncologic group than in the rTHA group (*p* =0 .176; risk 0.43) (*p* = 0.52; risk 0.74). Extended ileo-femoral approach and proximal femur replacement (*p* =0.02, risk ratio = 3.2; *p* = 0.04, rr = 2.1) were two significant independent predictors for dislocations, while belonging to group B (*p* = 0.04, rr = 2.6) was predictable for infections.

**Conclusion:**

Burch–Schneider-like antiprotrusio cages are a classical non-biological acetabular reconstruction method that surgeons should bear in mind when facing gross periacetabular bone losses, independently of their cause. However, dislocation and infection rates are high. Whenever possible, we suggest preserving the proximal femur in revision THA, and to use a less-invasive postero-lateral approach to reduce dislocation rates in non-oncologic cases.

**Supplementary Information:**

The online version contains supplementary material available at 10.1007/s00402-021-03929-6.

## Introduction

Revisions of total hip arthroplasty (rTHA) and periacetabular resections due to primary or secondary bone malignancies often pose surgeons to face the same problem, a major acetabular bone loss [1–3]. This is usually combined to poor quality of the surrounding pelvic bones becoming a true surgical challenge, particularly when bone loss results in compromise of acetabular column support. Several methods of acetabular reconstruction along with various biologic and non-biologic materials to supplement the periacetabular bone stock have already been described in literature, each one with its strengths and weaknesses [4, 5]. However, what finally drives the surgeon through the choice among different types of reconstruction techniques is the degree of the bone loss itself [2, 6]. Nowadays the customized triflange 3D printed acetabular components [7] or other custom-made prostheses [8, 9] are becoming more and more popular. However, Burch–Schneider-like antiprotrusio cages, combined or not with massive allograft or morselized grafts and/or cement, still remain a useful and relatively low-cost method in case of severe acetabular/periacetabular bone loss otherwise not bridgeable with simple cementless hemispherical trabecular metal cup [10–13]. Considering the fact that those cages do not rely upon a biological fixation but are simply mechanically fixed to the pelvis, there is certainly a risk for cage breakage and subsequent failure over time. This is even more likely in juvenile high-demand patients who face against periacetabular malignant bone tumors [13]. Furthermore, this is a complex surgery that often requires prolonged surgical time and large surgical exposures in patients with comorbidities related to their age or treated preoperatively with radio- and/or chemotherapy, with all the consequences that it carries in terms of intra- and post-operative complications. Indeed, due to the intra-pelvic extension of the malignancy or its closeness to the mayor iliac vessels as well as the presence of a cup protrusio or a huge intra-pelvic pseudotumor, surgical approaches could range from the classical postero-lateral approach to more invasive ones, such as extended Smith–Pethersen ilio-femoral approach or Enneking’s modified ilio-femoral approach. Those approaches are often more invasive for the surrounding soft tissues and they can lead to a higher short-term post-operative instability/dislocation rate irrespective of the type or the position of the prosthetic component used [10, 14].

Therefore, the aim of our study was to evaluate the clinical outcome, and estimate both the risk of cages’ failure and complications in a population of patients in which we implanted Burch–Schneider-like antiprotrusio cage due to failure of THA or periacetabular bone loss after resection of primary and secondary bone malignancies. Moreover, we investigated whether the surgical approach as well as the presence of a proximal femur replacement or the reason for implanting the antiprotrusio cage (revision THA versus oncologic surgery) could result a risk factor enhancing the chance of dislocation and/or infection and therefore predict the outcome of such complex patients.

## Patients and methods

### Patient selection

We retrospectively evaluated a cohort of 73 patients who have received a Burch–Schneider-like antiprotrusio cage from January 2008 to January 2018 due to periacetabular bone loss. Forty-five were females and 28 males with an average age of 64 years old (14–93) and average BMI (body mass index) of 24.9 (19–32). Patients were divided into two groups (group A and group B) based on the cause leading to the periacetabular bone defects.

Group A was made up of 40 oncological cases of which 22 had a primary pelvic bone tumor and 18 an acetabular/periacetabular bone metastases. Enneking e Dunham classification was used to describe the entity of bone defect related to the oncological resection [15]. Seventeen cases underwent a resection type II, 22 type I–II–III resections and one type II–III resection (Table 1).

**Table 1:** Demographic characteristics of Group A: periacetabular bone defects due to primary pelvic bone tumor or bone metastases

Patient number	Age (years)	Histotype	Enneking e Dunham	Follow-up (months)	RF survival (months)	Cotile	PFR	Surgical approach	Graft used
1	60	Renal cancer mtx	Type 1–2–3	86	86	DM	N	Extended ileo-femoral	Massive allograft
2	64	Renal cancer mtx	Type 2	35	35	DM	N	Extended postero-lateral	None
3	59	GII chondrosarcoma	Type 1–2–3	79	79	TRC	N	Extended ileo-femoral	Massive allograft
4	33	Leiomyosarcoma	Type 2–3	41	41	TRC	N	Extended ileo-femoral	None
5	33	GII chondrosarcoma	Type 1–2–3	113	113	TRC	N	Extended ileo-femoral	Massive allograft
6	16	Ewing sarcoma	Type 1–2–3	37	37	TRC	N	Extended ileo-femoral	Massive allograft
7	48	Thyroid cancer mtx	Type 1–2–3	36	36	TRC	Y	Extended ileo-femoral	Massive allograft
8	67	GII chondrosarcoma	Type 1–2–3	74	74	DM	N	Extended ileo-femoral	Massive allograft
9	66	Hemangiothelioma	Type 1–2–3	72	72	TRC	N	Extended ileo-femoral	Massive allograft
10	67	GII chondrosarcoma	Type 1–2–3	30	30	TRC	N	Extended ileo-femoral	Massive allograft
11	66	GI chondrosarcoma	Type 1–2–3	83	83	TRC	N	Extended ileo-femoral	Massive allograft
12	51	Breast cancer mtx	Type 2	36	36	DM	N	Extended postero-lateral	None
13	60	Dedifferentiated chondrosarcoma	Type 1–2–3	90	1	DM	Y	Extended ileo-femoral	Massive allograft
14	61	Chondrosarcoma grade.II	Type 1–2–3	86	86	TRC	N	Extended ileo-femoral	Massive allograft
15	55	Dedifferentiated chondrosarcoma	Type 1–2–3	33	33	TRC	N	Extended ileo-femoral	Massive allograft
16	45	Giant cell tumor	Type 1–2–3	132	132	DM	N	Extended ileo-femoral	Massive allograft
17	67	Leiomyosarcoma	Type 1–2–3	36	36	TRC	N	Extended ileo-femoral	Massive allograft
18	71	Breast cancer mtx	Type 2	75	75	DM	N	Extended postero-lateral	None
19	65	GII chondrosarcoma	Type 1–2–3	47	47	TRC	N	Extended ileo-femoral	Massive allograft
20	62	GII chondrosarcoma	Type 1–2–3	41	41	TRC	N	Extended ileo-femoral	Massive allograft
21	30	Malignant peripheral nerve sheath tumor	Type 1–2–3	36	36	TRC	N	Extended ileo-femoral	Massive allograft
22	14	Ewing sarcoma	Type 1–2–3	118	118	TRC	N	Extended ileo-femoral	Massive allograft
23	38	GII chondrosarcoma	Type 1–2–3	56	56	DM	N	Extended ileo-femoral	Massive allograft
24	53	Multiple myeloma	Type 2	36	36	DM	N	Extended postero-lateral	None
25	67	Osteoblastoma	Type 1–2–3	46	46	TRC	N	Extended ileo-femoral	Massive allograft
26	46	Breast cancer mtx	Type 1–2–3	30	30	TRC	N	Extended ileo-femoral	Massive allograft
27	65	Breast cancer mtx	Type 2	38	38	DM	N	Extended postero-lateral	None
28	74	Renal cancer mtx	Type 1–2–3	49	49	DM	N	Extended ileo-femoral	Massive allogrft
29	81	Carcinoma of unknown primary mtx	Type 2	36	36	DM	N	Extended postero-lateral	None
30	83	Multiple myeloma	Type 2	38	38	DM	N	Extended ileo-femoral	None
31	85	Prostate cancer mtx	Type 2	36	36	DM	N	Extended ileo-femoral	None
32	66	Breast cancer mtx	Type 2	36	36	DM	N	Extended ileo-femoral	None
33	42	Melanoma mtx	Type 2	36	1	DM	N	Extended postero-lateral	None
34	75	Breast cancer mtx	Type 2	36	36	DM	N	Extended ileo-femoral	None
35	71	Renal cancer mtx	Type 2	36	36	DM	N	Extended ileo-femoral	None
36	67	Breast cancer mtx	Type 2	44	15	TRC	Y	Extended postero-lateral	None
37	59	Breast cancer mtx	Type 2	36	36	DM	N	Extended ileo-femoral	None
38	59	Multiple myeloma	Type 2	39	10	DM	N	Extended ileo-femoral	CABG
39	66	GII chondrosarcoma	Type 2	39	39	DM	N	Extended ileo-femoral	None
40	76	Epithelioid angiosarcoma	Type 2	40	40	DM	Y	Extended postero-lateral	None

*DM* dual mobility, *TRC* total retention cup, *CABG* corticocancellous allogenic bone graft, *PFR* proximal femur replacement, *mtx* metastasis, *RF* *survival* revision free survival (considering any surgery in which at least one component of the implanted prostheses was exchanged)

Group B consisted of 33 patients with a periacetabular bone defect due to failure of THA. The causes of failure were aseptic loosening and THA infection in 24 and 9 cases, respectively. Ten of those patients had a history of hip dysplasia and they had already undergone multiple hip surgeries. In those non-oncological cases, we classified periacetabular bone losses according to Paprosky classification [16]. In eight cases, we found a pelvic discontinuity, nine were 3A type bone loss, five 3B, seven 2B and three cases 2C type bone loss (Table 2).

**Table 2:** Demographic characteristics of Group B: periacetabular bone defects due to failure of THA

Patient number	Age (years)	Cause	Paprosky	Follow-up (months)	RF survival (months)	Cotile	PFR	Surgical Approach	Graft used
1	72	Aseptic loosening	3a	22	22	DM	Y	Extended ileo-femoral	C.A.B.G
2	62	Cup protrusio	Pd	50	38	DM	N	Extended ileo-femoral	C.A.B.G
3	76	Aseptic loosening	2b	87	87	DM	N	Extended postero-lateral	None
4	43	Endopelvic pseudotumor	Pd	132	6	DM	N	Extended ileo-femoral	C.A.B.G
5	80	Aseptic loosening	Pd	41	41	TRC	N	Extended ileo-femoral	C.A.B.G
6	76	Aseptic loosening	Pd	44	44	TRC	Y	Extended ileo-femoral	C.A.B.G
7	72	Aseptic loosening	Pd	82	82	DM	Y	Extended ileo-femoral	Massive allograft
8	79	Aseptic loosening	2c	46	46	TRC	N	Extended postero-lateral	None
9	45	Aseptic loosening	Pd	108	108	DM	N	Extended postero-lateral	None
10	84	Aseptic loosening	3b	98	98	DM	N	Extended postero-lateral	C.A.B.G
11	91	Infection	3a	52	52	TRC	N	Extended postero-lateral	None
12	63	Aseptic loosening	2b	74	74	DM	N	Extended postero-lateral	C.A.B.G
13	48	Aseptic loosening	2b	92	92	DM	N	Extended postero-lateral	None
14	79	Infection	3b	86	86	TRC	Y	Extended postero-lateral	None
15	78	Aseptic loosening	2b	105	35	DM	N	Extended postero-lateral	C.A.B.G
16	69	Infection	3a	97	87	DM	Y	Extended postero-lateral	None
17	76	Aseptic loosening	2c	116	24	TRC	N	Extended postero-lateral	None
18	83	Infection	3a	36	36	DM	Y	Extended ileo-femoral	None
19	63	Infection	3a	137	137	DM	Y	Extended postero-lateral	None
20	83	Infection	3a	79	79	TRC	Y	Extended postero-lateral	None
21	63	Aseptic loosening	3a	94	94	DM	N	Extended ileo-femoral	None
22	59	Infection	3b	132	25	TRC	Y	Extended ileo-femoral	C.A.B.G
23	76	Cup protrusio	Pd	76	76	DM	Y	Extended ileo-femoral	None
24	57	Aseptic loosening	Pd	78	67	DM	N	Extended ileo-femoral	C.A.B.G
25	93	Aseptic loosening	Pd	41	1	TRC	N	Extended postero-lateral	None
26	60	Infection	3b	132	132	DM	N	Extended postero-lateral	None
27	78	Aseptic loosening	2b	94	94	DM	Y	Extended postero-lateral	C.A.B.G
28	67	Infection	3a	57	57	DM	N	Extended ileo-femoral	None
29	72	Aseptic loosening	2b	36	36	DM	N	Extended postero-lateral	None
30	76	Aseptic loosening	3a	39	39	DM	N	Extended ileo-femoral	C.A.B.G
31	61	Aseptic loosening	2b	47	47	DM	Y	Extended ileo-femoral	None
32	62	Aseptic loosening	3b	36	36	DM	Y	Extended postero-Lateral	None
33	74	Aseptic loosening	2c	38	38	DM	N	Extended postero-Lateral	None

*Pd* pelvic discontinuity, *THA* total hip arthroplasty, *DM* dual mobility, *TRC* total retention cup, *C.A.B.G.* corticocancellous allogenic bone graft, *RF* *survival* revision free survival (considering any surgery in which at least one component of the implanted prostheses was exchanged)

Inclusion criteria were the following: minimum follow-up of 2 year; Paprosky ≥ 2B type bone loss or Enneking type II, type II–III and type I–II–III localization of primary/secondary bone tumors; implantation of an acetabular antiprotrusio cage “Burch–Schneider-like”; second-stage reimplantation in patients with a previous history of periprosthtic infection who already underwent the first-stage treatment with removal of the prosthesis, implantation of an antibiotic-loaded cement spacer, e.v. prolonged antibiotic treatment and who had been tested and excluded (blood test: CRP, ESR; joint aspiration and culture) for any presence of active/recurrent infection at the time of the index surgery.

We excluded patients who underwent an implantation of any other devices to by-pass the acetabular/periacetabular bone loss rather than the “Burch–Schneider-like” antiprotrusio cage (Muller-type rings, Ganz-type cages, trabecular-metal cup-cage reconstructions, jumbo cups, customized triflange acetabular components, etc.) and patients with a follow-up less than 24 months. Other exclusion criteria were the following: patients with an active periprosthetic infection or patients who resulted infected at the intra-operative cultures at the time of a second-stage reimplantation due to a previous periprosthetic infection; failures of TKA with a periacetabular bone loss less than type 2B according to Paprosky.

### Surgical approach and implant type

The decision-making on surgical approach was based on many factors including intra-pelvic extension of the bone tumor, the closeness of the malignancy to the iliac vessels, the presence of a huge intra-pelvic pseudotumor THA related as well as a pelvic discontinuity associated to an intra-pelvic cup protrusio. In revision THA, whenever possible, we performed the same surgical approach utilized during the first/previous surgeries. Thus, in 45 patients, we used an extended ileo-femoral approach (four extended Smith–Pethersen ilio-femoral approaches and 41 Enneking’s modified ilio-femoral approaches) while the remaining 28 patients underwent an extended postero-lateral approach.

For every patient, the bone loss was by-passed through the use of an acetabular antiprotrusio cage: 64 partial pelvic replacement (PPR) cages (Waldemar Link GmbH, Hamburg, Germany) and nine Burch–Schneider reinforcement cages (Zimmer Biomet, Warsaw, IN, USA). The associated cemented acetabular cup component within the cage was a self-retaining liner in 27 cases [13 jump system (Permedica S.p.a., Merate, LC, Italy); 9 UHMWPE cemented acetabular retention cup (Groupe Lépine, Genay, France), 3 lubinus polyethylene acetabular cup (Waldemar Link GmbH, Hamburg, Germany), 2 freedom constrained acetabular liners (Zimmer Biomet, Warsaw, IN, USA)] and a double mobility cup in 46 patients [33 Active Articulation Avantage (Zimmer Biomet, Warsaw, IN, USA), 13 BiMobile (Waldemar Link GmbH, Hamburg, Germany)].

Moreover, in addition to the antiprotrusio cage, to fulfill the associated bone loss, we utilized only cement in 38 cases while in other 35 cases, an allogenic bone bank graft was chosen (corticocancellous allogenic bone graft was used in 13 patients, while in 22 cases with a type I–II–III bone tumor extension and in two cases of pelvic discontinuity associated to cup protrusio, we utilized a massive hemipelvic allograft stabilized with screws or plates and screws to the pubic symphysis and the sacroiliac joint). To enhance primary stability, we added a reinforcement mesh in between the neo-acetabulum and the proximal femur in five cases of which three Trevira tube polyethylene terephthalate (Implantcast Gmbh, Buxtehude, Germany) and two ligament advanced reinforcement system—LARS (surgical implants and devices, Arc-sur-Tille, France). Furthermore, four cases of group A and 13 cases of group B underwent a contemporary proximal femoral replacement Megasystem-C^®^ (Waldemar Link GmbH, Hamburg, Germany).

### Clinical and radiological evaluations

All patients were evaluated pre-operatively by standard radiology (AP and axial hip view) and computerized tomography (CT) scan. Moreover, for patients with bone tumors, a magnetic resonance imaging (MRI) was always performed to evaluate the local extension of the disease and consequently plan the bone resection. Regarding the revision arthroplasty group, independently from the presence of a previous history of periprosthetic infection, every patient was screened both through blood examinations (C-reactive protein—CRP, erythrocyte sedimentation rate—ESR, white blood cell—WBC) and by a joint aspiration (for both bacterial culture and assessment of synovial with blood cell count, synovial neutrophil percentage and leukocyte esterase) to rule out any septic condition.

Patients of the revision arthroplasty group were both clinically and radiologically followed up at 30 days, 3 months, 6 months and then yearly, while oncological patients were followed up based on their histological diagnosis. Pre-operatively and then at every clinical evaluation, the modified Harris hip score [17] and the presence of a Trendelenburg gait ware determined for both group of patients. Moreover, during the same review, patients were assessed for radiographic complications (including loosening/cage migration, screw breakage, plate fracture) [18], but only those ones leading a subsequent reoperation were recorded.

### Statistical analyses

Statistical analysis was performed using SPSS® statistics software (IBM®, Armonk, New York, USA) and R Studio statistical software V1.3.159 (R-Studio PBC, Boston, Massachusetts, USA). Demographical data such as age and BMI were tested for the normal distribution using the Kolmogorov–Smirnov (K-S) test. The Student t-test was used to compare pre- and post-operative HSS results, while the chi-square test was used to compare pre- and post-operative presence of Trendelemburg gait, both taking a *p* values < 0.05 to be statistically significant. In addition, the correlation between Trendelemburg gait and dislocation was performed using the chi-square test taking *p* < 0.05 as statistically significant. In case of the > 20% of cells in a 2 × 2 table have expected to count less than 5, a 2-sided Fisher exact test was performed. To take account of the competing risk of death we calculated the cumulative incidence function (CIF) [19], by using the method proposed by Gray [20]. To estimate both the risk of failure and complication among subjects receiving surgery for oncologic reason compared to those receiving surgery for rTHA, we used the proportional subdistribution hazards regression model described in Fine and Gray [21]. The Cox proportional-hazards regression model was utilized to identify multivariate risk factors predictive of instability and infection [independent variables included the surgical approach used as well as the presence of a proximal femur replacement and the reason for implanting the antiprotrusio cage (revision THA versus oncology surgery)], with the risk ratio and 95% confidence interval used to measure the strength of the association [22].

## Results

### Clinical evaluation

The average follow-up of the revision THA group was 86 months (24–137 months), while for the oncologic patient group was 74 months (24–122 months). Twenty-one (28.7%) patients died. The majority of them belonged to the oncologic group where the deaths were 14/40 (35%) of which nine in patients with secondary bone metastases and five in patients with primary oncological bone disease. The histotypes of primary bone tumors in patients who died of disease progression were: spindle cell sarcoma, Ewing sarcoma, dedifferentiated chondrosarcoma, G2 chondrosarcoma and malignant peripheral nerve sheath tumor MPNST (Table 1). Among the revision arthroplasty group, 7 (21%) patients died due to causes other than the hip revision surgery.

Demographic data, such as age and BMI, were normally distributed (age: K–S = 0.153; *p* = 0.572; skewness = − 0.969; kurtosis = 1.136) (BMI: K–S = 0.130; *p* = 0.060; skewness = − 0.559; kurtosis = − 1.06).

The modified Harris hip score showed a significant increase pre- to post-operatively (*p* < 0.05): an average pre-operative score of 14 (14–32) to an average score of 61 (28–92) at the last follow-up (*p* < 0.05) and it was substantially superimposable within the two groups (*p* > 0.05). We recorded a Trendelemburg gait in 18 patients (24%) with a significant difference between pre- and post-operative findings (*χ*^2^ test = 9.56; *p* = 0.002).

We found no association between post-operative Trendelemburg gait and dislocations (*χ*^2^ test = 0.821, Fisher exact test = 0.532; *p* = 0.365).

### Survivorship analysis

**Fig. 1:**
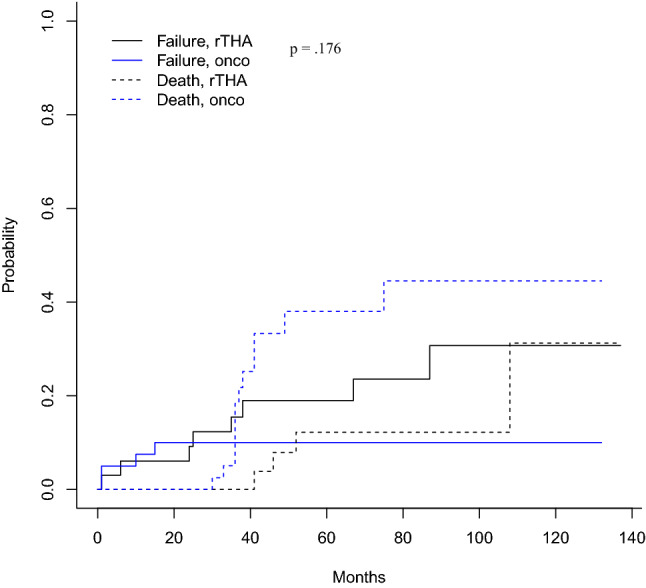
Cumulative incidence function for cages’ failure taking into account of the competing risk of death

**Fig. 2:**
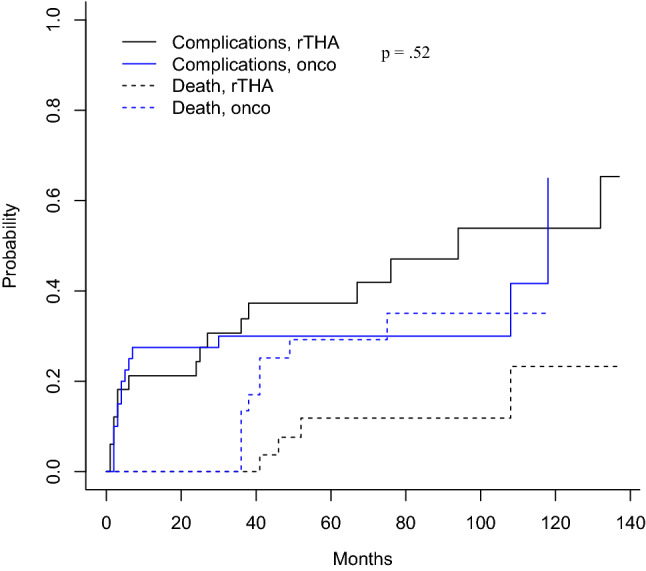
Cumulative incidence function for complications taking into account of the competing risk of death

Taking into account the competing risk of death, the estimates of risk of failure at 5 years were 0.1 and 0.19 for the oncologic and the rTHA group, respectively (Table 3, Fig. 1), while the risk of complication at 5 years was 0.3 and 0.37 for the oncologic and the rTHA group, respectively (Table 3, Fig. 2). Comparing both groups, the probability of failure was 57% lower in the oncologic group than in the rTHA group without reaching the statistical significance [*p* = 0.176; risk 0.43 (CI 0.13–1.42)], while the probability of complications was 26% lower in the oncologic group than in the rTHA group [*p* = 0.52; risk 0.74 (CI 0.37–1.46)] (Table 3).

**Table 3:** Competing risk analysis for failure and complications

Competing risk analysis for failure	Time (months)						*p* value
	20	40	60	80	100	120	
Revision THAs for failure	0.060	0.189	0.189	0.235	0.306	0.306	0.176
Oncologic resections for failure	0.100	0.100	0.100	0.100	0.100	0.100	
Revision THAs for competing death	0.000	0.000	0.122	0.122	0.122	0.312	0.011
Oncologic resections for competing death	0.000	0.251	0.379	0.444	0.444	0.444	
**Competing risk analysis for complications**							
Revision THAs for failure	0.212	0.373	0.373	0.470	0.539	0.539	0.522
Oncologic resections for failure	0.275	0.300	0.300	0.300	0.300	0.300	
Revision THAs for competing death	0.000	0.000	0.118	0.118	0.118	0.232	0.062
Oncologic resections for competing death	0.000	0.169	0.292	0.350	0.350	0.350

The Cox proportional hazards regression analyses revealed that the surgical approach used, the presence of a proximal femur replacement and the reason for implanting the cage were significant independent predictor, respectively, for post-operative instability the former two (*p* = 0.02, risk ratio = 3.2; *p* = 0.04, risk ratio = 2.1) and infections the latter (*p* = 0.04, risk ratio = 2.6). Specifically, the odds of instability were estimated to be three times higher the 1st year for patients who were treated with an extended ileo-femoral approach and two times higher for those who underwent a complementary proximal femur replacement, as well as the odds of infections were estimated to be more than two times higher for the group in which the reason for implanting a cage was due to revision THA (Table 4).

**Table 4:** Predictor of dislocation and infection after implanting the antiprotrusio cage

	Dislocation			Infection		
	*p* value	Odds ratio	95% CI	*p* value	Odds ratio	95% CI
**Surgical approach**						
Postero-lateral approach	0.08	0.9	0.6–1.2	0.48	0.76	0.6–0.9
Extended ilio-femoral approach	0.02	3.2	2.4–3.6	0.12	0.97	0.9–1.3
Proximal femur replacement	0.04	2.1	1.9–2.6	0.64	0.78	0.7–1.0
**Reason for implanting an antiprotrusio cage**						
Revision THAs	0.36	0.8	0.5–1.3	0.04	2.6	1.9–3.1
Oncological resections	0.09	1.2	0.7–1.5	0.18	1.4	1.1–1.6

### Complications and failures

A total of 46 complications were observed in 31 patients (42%): 24 dislocations (18 primary and six recurrent dislocations), eight peroneal nerve palsies (six transients with full recovery and two persistent; every of those were related to dislocations except one persistent palsy related to the index surgery), ten infections, three aseptic loosening and one local recurrence of the underling oncological disease. Details of complications per patient are reported in Supplementary Material as Tables 5, 6 (the full report of complications is also extensively described in Supplementary Material).

## Discussion

One of the aims of the study was to investigate whether this non-biological type of acetabular reconstruction could keep its mechanical strength with time at mid/long-term follow-up, independently of the nature of acetabular/periacetabular bone loss. Taking into account the competing risk of death, in our series, the risk of failure was 30% and 10% at 10 years for the rTHA and oncologic group, respectively, with no differences between the two groups.

The first long-term cages’ survival rate was reported by Berry and Müller [23] in which they found 76% survival rate in 32 hips at 5 years of follow-up. More recently, in the review published by Aprato et al. [24], the survival rate of those particular type of reinforcement rings among 13 different articles ranges from 72 to 100% at a medium follow-up of 5.6 years.

Rowell et al. [25] were the first to report the treatment of 47 acetabular bone metastasis with the use of PPR link system between 2006 and 2017. They found a surprisingly high survivorship of 91% free from all-cause revision or reoperation, probably due to the short survival expectancy of metastatic patients.

Focusing on structural/mechanical failure of the cage itself (aseptic loosening/fixation failure/cage breakage), Hsu et al. [14] described a total of six (19.4%) failures with associated component migration at the latest follow-up (10 years), reporting that three implants had broken iliac screws but no cage breakages occurred. Among our series, we reported only four cases (5.5%) of structural/mechanical failure that required a re-intervention equally distributed between the two groups.

Udomkiat et al. [26] retrospectively reviewed a series of 18 Burch–Schneider cages and found a 6-year survivorship for mechanical failure of 63.6% including both cages revised for aseptic loosening and those that had radiographic loosening but had not been revised.

In line with the previously reported literature [11, 23, 25, 27–31], we also observed good functional results with a median modified Harris hip score of 61 and no significative differences among the two groups. Nevertheless, we reported a high incidence of Trendelenburg gait (24%) among both groups, but we found no association in between this particular type of complication and the post-operative dislocation rate (*F* = 0.532). Differently from our report, Udomkiat et al. found that 38.8% (7 out of 18 patients) of their cages’ series had a dislocation. They described that the dislocation rate was related to muscle weakness (*p* = 0.028).

Among our study population, we reported a high incidence of complications. Almost half of our patients had at least one complication (42%). Taking into account the competing risk of death, the risk of getting complication was 53% and 30% at 10 years for the rTHA and oncologic group, respectively, with no differences among the groups. The most common complication was dislocation (primary dislocation in 24.7% of patients) followed by infection (13.7% of patients).

In our study, we observed the surgical approach/exposure along with the presence of a complementary proximal femur replacement as the two major independent risk factors for dislocation. Indeed, the odd ratio of dislocation resulted three times higher (*p* = 0.02, risk ratio = 3.2) in those patients who underwent an extended ileo-femoral approach and two times higher (*p* = 0.04, risk ratio = 2.1) for those who also had a proximal femur replacement. This can be related on the fact that this surgical approach was most frequently used in oncologic or challenging cases, in the presence of huge endopelvic pseudotumor in contact with iliac vessels or endopelvic cup protrusion associated with pelvic discontinuity, in which a postero-lateral approach could not be the first choice. Moreover, all the patients (100%) of the rTHA group who got a dislocation underwent also a proximal femur replacement and, actually, every patient of the oncologic group who had a proximal femur replacement (four patients) got a dislocation as well, independently of the surgical approach used. Based on the fact that during the implantation of a proximal femur replacement prostheses both the gluteus muscles and the psoas muscle have to be detached, it also remarks the importance that soft tissues play in the stability of a revision THA. It means that both factors, an extensive invasive surgical approach and the presence of a proximal femur replacement, could justify the reason why we had that high rates of dislocation in both groups (dislocation rate: 35% in group A and 30.3% in group B).

A lower dislocation rate was reported in the periacetabular metastatic series of patients by Rowell et al. [25] who described four events in 46 patients (9%). A slightly higher rate of dislocation (17%) was described by Mark Clayer [12] in a series of 29 patients undergoing implantation of an anti-protrusio cage for metastatic pelvic disease. Such low dislocation rates could be related to the short survival of metastatic patients. Indeed, in the latter report, the median length of patient survival was only 12 months (3 days–100 months) after the procedure.

Regarding the second-most common complication, we found that implanting an anti-protrusio-cage due to failure of a previous THA or revision THA was an independent risk factor in predicting post-operative infections. Indeed, the infection rate among the two groups was, respectively, 2.5% and 24.2% in the oncologic group and revision THA group and the estimated odds ratio of infections was more than two times higher (*p* = 0.04, risk ratio = 2.6) for the latter group. Despite no previous study has compared those two different categories of patients, single literature reports are in line with our findings concerning the oncologic group (percentage of infection ranging from 2 to 8%) [12, 25, 27] but the infection rates among the revision THA are reported to be lower than what we have found (percentage of infection ranging from 3 to 10%) [14, 30, 31]. We believe that higher infection rate in our revision THR series can be related to previous multiple surgeries, with an average of 2.9 procedures before the index operation.

This study has several limitations. We studied two non-homogeneous groups of patients, but our initial purpose was to evaluate the durability of a non-biologic implant in bypassing a gross acetabular/periacetabular bone defect independently of the cause of the bone loss itself. Furthermore, the two groups have in common a poor host bone healing potential, either because of the scarce quality of bone that sustained many previous surgeries [32] or because the bone lesion (e.g. metastasis) is not expected to heal [33] or because the periacetabular bone resection (e.g. primary bone tumors) has been bypassed with a massive allograft [34] that has no capacity to support the bone growth over an ingrowth material (e.g. trabecular metal).

Second, the sample size of our study is not big enough to give a proper consistency at the statistical analysis performed, therefore the interpretation of data cannot be taken as milestone.

Finally, despite we performed X-rays at every out-patient control, we have only recorded those cases that required a subsequent revision surgery due to cage loosening/migration/structural breakage. This could therefore lead to an underestimation of intrinsic cages’ mechanical failures.

As far as we know, there are no previously reported studied in literature comparing those two groups of patients with the purpose to evaluate the potentialities and weaknesses of this particular bridging acetabular cage implant, independently of the nature causing the acetabular/periacebular bone defect as well as to investigate whether or not there could exist some difference in complication rates among the two different groups and if those could be related to some specific risk factors.

## Conclusion

Burch–Schneider-like antiprotrusio cages are useful implants when facing against different types of gross acetabular/periacetabular bone losses, with an overall high long-term survival rate. However, complication rate is high, mostly dislocations and infections. Whenever possible, we suggest both to spare the proximal femur along with its muscular insertions in rTHA, and to use a less-invasive postero-lateral approach to reduce dislocation rates.

## Supplementary Information

Below is the link to the electronic supplementary material.

Supplementary file1 (DOCX 15 kb)

Supplementary file2 (DOCX 20 kb)

Supplementary file3 (DOCX 18 kb)

## References

[1] Brown TS, Salib CG, Rose PS (2018). Reconstruction of the hip after resection of periacetabular oncological lesions: a systematic review. Bone Jt J.

[2] Sheth NP, Nelson CL, Springer BD (2013). Acetabular bone loss in revision total hip arthroplasty: evaluation and management. J Am Acad Orthop Surg.

[3] Beadel GP, McLaughlin CE, Wunder JS (2005). Outcome in two groups of patients with allograft-prosthetic reconstruction of pelvic tumor defects. Clin Orthop Relat Res.

[4] Fryhofer G, Ramesh S, Sheth N (2019) Acetabular reconstruction in revision total hip arthroplasty. J Clin Orthop Trauma 11. 10.1016/j.jcot.2019.11.004 10.1016/j.jcot.2019.11.004 PMC6985018 32001979

[5] Gamradt SC, Lieberman JR (2003). Bone graft for revision hip arthroplasty: biology and future applications. Clin Orthop Relat Res.

[6] Shon W, Santhanam S, Choi J (2016). Acetabular reconstruction in total hip arthroplasty. Hip Pelvis.

[7] De Martino I, Strigelli V, Cacciola G (2019). Survivorship and clinical outcomes of custom triflange acetabular components in revision total hip arthroplasty: a systematic review. J Arthroplasty.

[8] Müller PE, Dürr HR, Wegener B (2002). Internal hemipelvectomy and reconstruction with a megaprosthesis. Int Orthop.

[9] Rudert M, Holzapfel BM, Pilge H (2012). Partial pelvic resection (internal hemipelvectomy) and endoprosthetic replacement in periacetabular tumors. Oper Orthop Traumatol.

[10] Pieringer H, Auersperg V, Böhler N (2006). Reconstruction of severe acetabular bone-deficiency. The Burch–Schneider antiprotrusio cage in primary and revision total hip arthroplasty. J Arthroplasty.

[11] Regis D, Sandri A, Bonetti I (2014). Acetabular reconstruction with the Burch–Schneider antiprotrusio cage and bulk allografts: minimum 10-year follow-up results. Biomed Res Int.

[12] Clayer M (2010). The survivorship of protrusio cages for metastatic disease involving the acetabulum. Clin Orthop Relat Res.

[13] Li D, Guo W, Yang R (2011). Utilization of reinforced acetabular cages with caudal flange in reconstructing pelvic defect after acetabular tumor resection. Zhongguo Xiu Fu Chong Jian Wai Ke Za Zhi.

[14] Hsu CC, Hsu CH, Yen SH, Wang JW (2015). Use of the Burch–Schneider cage and structural allografts in complex acetabular deficiency: 3- to 10-year follow up. Kaohsiung J Med Sci.

[15] Enneking WF, Dunham WK (1978). Resection and reconstruction for primary neoplasms involving the innominate bone. J Bone Jt Surg Ser A.

[16] Paprosky WG, Perona PG, Lawrence JM (1994). Acetabular defect classification and surgical reconstruction in revision arthroplasty. A 6-year follow-up evaluation. J Arthroplasty.

[17] Kumar P, Sen R, Aggarwal S (2019). Reliability of modified Harris hip score as a tool for outcome evaluation of total hip replacements in Indian population. J Clin Orthop Trauma.

[18] Johnston RC, Fitzgerald RH, Harris WH (1990). Clinical and radiographic evaluation of total hip replacement. A standard system of terminology for reporting results. J Bone Jt Surg Ser A.

[19] Gillam MH, Ryan P, Graves SE (2010). Competing risks survival analysis applied to data from the Australian orthopaedic association national joint replacement registry. Acta Orthop.

[20] Robert JG (1988) A class of K-sample tests for comparing the cumulative incidence of a competing risk. Ann Stat 16(3):1141–1154

[21] Fine JP, Gray RJ (1999). A proportional hazards model for the subdistribution of a competing risk. J Amn Stat Assoc.

[22] Cox DR (1972). Regression models and life-tables. J R Stat Soc B.

[23] Berry DJ, Muller ME (1992). Revision arthroplasty using an anti-protrusio cage for massive acetabular bone deficiency. J Bone Jt Surg Ser B.

[24] Aprato A, Olivero M, Vergano LB, Massè A (2019). Outcome of cages in revision arthroplasty of the acetabulum: a systematic review. Acta Biomed.

[25] Rowell P, Lowe M, Sommerville S, Dickinson I (2019). Is an acetabular cage and cement fixation sufficiently durable for the treatment of destructive acetabular metastases?. Clin Orthop Relat Res.

[26] Udomkiat P, Dorr LD, Won YY (2001). Technical factors for success with metal ring acetabular reconstruction. J Arthroplasty.

[27] Hoell S, Dedy N, Gosheger G (2012). The Burch–Schneider cage for reconstruction after metastatic destruction of the acetabulum: outcome and complications. Arch Orthop Trauma Surg.

[28] Peters CL, Curtain M, Samuelson KM (1995). Acetabular revision with the Burch–Schnieder antiprotrusio cage and cancellous allograft bone. J Arthroplasty.

[29] Gill TJ, Sledge JB, Müller ME (1998). The Burch–Schneider anti-protrusio cage in revision total hip arthroplasty. Indications, principles and long-term results. J Bone Jt Surg Ser B.

[30] Regis D, Sandri A, Bonetti I (2012). A minimum of 10-year follow-up of the Burch–Schneider cage and bulk allografts for the revision of pelvic discontinuity. J Arthroplasty.

[31] Goodman S, Saastamoinen H, Shasha N, Gross A (2004). Complications of ilioischial reconstruction rings in revision total hip arthroplasty. J Arthroplasty.

[32] Gaiani L, Bertelli R, Palmonari M, Vicenzi G (2009). Total hip arthroplasty revision in elderly people with cement and Burch–Schneider anti-protrusio cage. Chir Organi Mov.

[33] Yazawa Y, Frassica FJ, Chao EYS (1990). Metastatic bone disease: a study of the surgical treatment of 166 pathologic humeral and femoral fractures. Clin Orthop Relat Res.

[34] Campanacci D, Chacon S, Mondanelli N (2012). Pelvic massive allograft reconstruction after bone tumour resection. Int Orthop.

