# Supplementary material for: The use of a non-biological, bridging, antiprotrusio cage in complex revision hip arthroplasty and periacetabular reconstructive oncologic surgery. Is still today a valid option?: A mid/long-term survival and complications’ analysis
Source: Arch Orthop Trauma Surg. 2021 May 24;142(4):681–90. doi: 10.1007/s00402-021-03929-6 (PMC8924141; doi:10.1007/s00402-021-03929-6)
Supplement: Supplementary file 1 — Supplementary file1 (DOCX 15 kb) [file 402_2021_3929_MOESM1_ESM.docx]

***Complications and Related Treatments***

Among the revision THA group (33 patients) we reported a total of 10 dislocations in 5 patients (5 primary and 5 recurrent dislocations). All of them had also a proximal femur replacement. Two cases occurred within the first month after surgery and they were treated with closed reduction and subsequent application of pelvic brace for six weeks. The other three cases underwent open surgical reduction. In one case, after multiple episodes of dislocation during the first month post-operatively, the femoral head was replaced with a longer one and greater anteversion was provided to the proximal component of the femoral megaprosthesis. After 6 months this patient experienced a recurrent dislocation that was treated surgically with the replacement of the previous implanted double motility cup with a constrained liner cup and associated tenotomy of the adductor muscles. A second patient presented a dislocation after two years from index surgery and he was treated by open reduction and replacement of the proximal component of the femoral megaprosthesis with a high-offset one and 5° of retroversion. Two months later, following a new dislocation, the double mobility cup was replaced by a constrained liner cup associated with capsule’s reconstruction with the use of LARS mesh. In the third case the dislocation occurred 6 months after surgery at it was treated with open reduction and replacement of the proximal component of the femoral megaprosthesis with a longer one (1 cm elongation) and 5° of retroversion. One month later this patient presented a new dislocation treated by replacing the proximal component of the femoral megaprosthesis with a new one with a cervical-diaphyseal angle of 135° associated to capsule’s reconstruction by the use of a synthetic mesh. After four months another recurrent dislocation occurred that was treated with replacement of the double mobility cup with a constrained liner cup and femoral head exchange from 22 to 28 mm. Finally, this patient had a new dislocation 3 years later treated with retention cup exchange with a new total retention cup and positioning of a new 126° cervical-diaphyseal angle proximal femoral component.

We observed 1 case of aseptic loosening of the cage itself and one case of aseptic loosening of the acetabular component within the cage. The first case occurred 27 months post-operatively and it was related to both periprosthetic proximal femur bone reabsorption and cage’s screw breakage with a supero-lateral migration (> 5 mm) of the cage. A new antiprotrusio-cage with a constrained liner cup was implanted and the femoral stem was revised with a cemented proximal femur megaprosthesis. The second case, 3 years after the index operation, presented a breakdown of the cement mantel interface within the cage and metal back of the double motility cup with subsequent cup loosening. A new cup was cemented inside of the cage and two new screws were placed on the cage’s ischial flange.

Finally, 8 septic complications occurred among this group. The first patient underwent a classical two-stage revision surgery with an antibiotic cement spacer and then the implantation of custom made triflange prosthesis. The second case also had a two-stage revision surgery with a final new cage re-implantation. The third case, a patient suffering from rheumatoid arthritis, underwent an antibiotic cement spacer implantation. The infection in this patient was then superimposed few months later by the ipsilateral total knee prosthesis infection, also treated with a cement spacer, and a contralateral THA infection 4 years later (waiting for explantation). The fourth patient was treated by a Girldestone procedure. Two more patients showed an acute infection (2 and 3 weeks after the index surgery respectively) and were successfully treated with debridement, antibiotics and implant retention (DAIR). Finally, 2 cases presented a superficial infection during the first month after surgery and they were both treated by washing, wound debridement and e.v. antibiotics (Table III). Only two of those patients (25%) who got an infection underwent the index surgical procedure due to a previous THA septic loosening.

Two post-operative transient peroneal nerve palsies occurred, both treated conservatively and recovered within 9 months.

The average number of surgical treatments received from patients in the revision THA group before the index antiprotrusio cage implantation was 2.92 per patient.

Among the oncologic group the complications were classified according to Henderson's classification[25]. The 14 dislocations, occurring in 13 patients (13 primary and 1 recurrent dislocations), were therefore classified as type 1A; Four of those cases had also a proximal femur replacement. 7 dislocations occurred 1, 2, 2, 4, 5, 9 months and two years after surgery respectively, they were treated by closed reduction and subsequent application of a pelvic brace for six weeks. Two other cases, presenting a dislocation two months after index surgery, were simply treated with open surgical reduction without any prostheses’ component exchange. Another case occurred one month post-operatively and it was treated surgically with open reduction and replacement of the proximal component of the femoral megaprostheses with a high-offset one associated with a reduced anteversion of 5°. Two more cases had a dislocation 1 months and 15 months respectively after the first surgery, treated in both cases with open reduction with femoral head exchange with a longer one (from small -4mm to x-large +8mm). The former also underwent a capsule’s reconstruction with a mesh. The last patient presented a dislocation two months post-operatively and he was treated with closed reduction and pelvic brace for six weeks. A recurrent dislocation occurred 10 months later, and it was surgically treated replacing the double motility cup with a constrained liner cup associated with capsule’s reconstruction with a tubular mesh.

In 5 of those dislocations a sciatic nerve palsy was reported after surgery; three were transient and therefore recovered within a year while 2 were persistent and did not recover.

Two infections were observed; one was classified as Henderson's type 1B or superficial infection (wound dehiscence). It occurred 20 days after surgery and it was successfully treated with lavage, debridement and e.v antibiotics. The other case, classified as 4B or deep infection, presented acutely 63 months post-operatively (late onset of an acute hematogenous infection) and it was successfully treated with DAIR.

We recorded one aseptic loosening among this group. It was a cage mobilization associated with screws breakage (classified as type 3A complication) occurred 108 months after index surgery. The cage was replaced with a new antiprotrusio cage associated to a cemented dual motility cup.

A recurrence of the oncologic disease (chondrosarcoma G2) was reported in one patient (type 5B complication) which determined the mobilization of the cage. This patient was treated with an external hemipelvectomy.
