# Supplementary material for: The use of a non-biological, bridging, antiprotrusio cage in complex revision hip arthroplasty and periacetabular reconstructive oncologic surgery. Is still today a valid option?: A mid/long-term survival and complications’ analysis
Source: Arch Orthop Trauma Surg. 2021 May 24;142(4):681–90. doi: 10.1007/s00402-021-03929-6 (PMC8924141; doi:10.1007/s00402-021-03929-6)
Supplement: Supplementary file 2 — Supplementary file2 (DOCX 20 kb) [file 402_2021_3929_MOESM2_ESM.docx]

| Patient Number | PFR | Time to complication  (months) | Treatment | Subsequent complications | Nrumber of subsequent complications | Treatment of subsequent complications* |
| --- | --- | --- | --- | --- | --- | --- |
|  |  |  | ***DISLOCATION*** |  |  |  |
| 1 | Y | 1 | Longer femoral Head and greater antiversion | Y | 1 | 1= 6m; Constrained Liner Cup |
| 2 | Y | 24 | High Offset PFR  5° Retroversion | Y | 1 | 1= 2m; DM+LARS |
| 3 | Y | 6 | 1cm Longer PFR  5°Retroversion | Y | 3 | 1= 1m, New PFR 135°  2= 4m; Constrained liner cup  3= 36m; New PFR 126° |
| 4 | Y | 1 | Pelvic brace for 6 weeks | N | - | - |
| 5 | Y | 1 | Pelvic brace for 6 weeks | N | - | - |
|  |  |  | ***ASEPTIC LOOSENING*** |  |  |  |
| 6 | N | 27 | New Cage+  Constrained Liner Cup | N | - | - |
| 7 | N | 36 | New cemented acetabular cup | N | - | - |
|  |  |  | ***INFECTION*** |  |  |  |
| 3 | Y | 96 | DAIR | N | - | - |
| 4 | Y | 38 | 2-stage revision  Custom made triflange | N | - | - |
| 8 | N | 35 | Spacer Cement | N | - | - |
| 9 | N | 67 | 2-stage revision  New cage | N | - | - |
| 10 | N | 106 | DAIR | N | - | - |
| 11 | N | 50 | Girldestone | N | - | - |
| 12 | N | 1 | Washing, Wound debridement, Antibiotics | N | - | - |
| 13 | N | 2 | Washing, Wound debridement, Antibiotics | N | - | - |
|  |  |  | ***NERVE PALSIE*** |  |  |  |
| 14 | N | *Postoperative* | *Conservative* | N | - | - |
| 15 | N | *Postoperative* | *Conservative* | N | - | - |

**Table V. Complications of rTHA group and related treatments**

*Number of subsequent complications = Months to subsequent complication; Type of treatment
