# Supplementary material for: The use of a non-biological, bridging, antiprotrusio cage in complex revision hip arthroplasty and periacetabular reconstructive oncologic surgery. Is still today a valid option?: A mid/long-term survival and complications’ analysis
Source: Arch Orthop Trauma Surg. 2021 May 24;142(4):681–90. doi: 10.1007/s00402-021-03929-6 (PMC8924141; doi:10.1007/s00402-021-03929-6)
Supplement: Supplementary file 3 — Supplementary file3 (DOCX 18 kb) [file 402_2021_3929_MOESM3_ESM.docx]

**Table VI. Complications of oncological group and related treatments**

| Patient Number | PFR | Henderson classification | Time to complication (m) | Treatment | Subsequent complications | Nrumber of subsequent complications | Treatment of subsequent complciations* |
| --- | --- | --- | --- | --- | --- | --- | --- |
|  |  |  |  | ***DISLOCATION*** |  |  |  |
| 1 | N | 1A | 1 | Close reduction  Pelvic brace for 6 weeks | N | - | - |
| 2 | Y | 1A | 2 | Close reduction  Pelvic brace for 6 weeks | N | - | - |
| 3 | N | 1A | 2 | Close reduction  Pelvic brace for 6 weeks | Y | 1 | 1= 10m; Constrained Liner Cup |
| 4 | Y | 1A | 4 | Close reduction  Pelvic brace for 6 weeks | N | - | - |
| 5 | Y | 1A | 5 | Close reduction  Pelvic brace for 6 weeks | N | - | - |
| 6 | N | 1A | 9 | Close reduction  Pelvic brace for 6 weeks | N | - | - |
| 7 | N | 1A | 24 | Close reduction  Pelvic brace for 6 weeks | N | - | - |
| 8 | N | 1A | 2 | Open reduction | N | - | - |
| 9 | N | 1A | 2 | Open reduction | N | - | - |
| 10 | N | 1A | 1 | High Offset PFR  More Retroversion(5°) | N | - | - |
| 11 | N | 1A | 10 | Longer Femoral head | N | - | - |
| 12 | N | 1A | 15 | Longer Femoral head | N | - | - |
| 13 | Y | 1A | 2 | Close reduction  Pelvic brace for 6 weeks | N | - | - |
|  |  |  |  | ***ASEPTIC LOOSENING*** |  |  |  |
| 14 | N | 3A | 108 | New Antiprotrusio Cage with DM cemented | N | - | - |
|  |  |  |  | ***INFECTION*** |  |  |  |
| 15 | N | 4A | 1 | Washing, debridement, Antibiotics | N | - | - |
| 16 | N | 4A | 1 | Washing, debridement, Antibiotics | N | - | - |
| 17 | N | 4B | 38 | 2-stage revision  Custom made triflange | N | - | - |
|  |  |  |  | ***RECURRENCE OF DISEASE*** |  |  |  |
| 17 | N | 5A | 33 | External hemipelvectomy | N | - | - |

*Number of subsequent complications = Months to subsequent complication; Type of treatment
